# Oral care quality—Do humanity aspects matter? Nursing staff's and older people's perceptions

**DOI:** 10.1002/nop2.461

**Published:** 2020-02-26

**Authors:** Maria Andersson, Bodil Wilde‐Larsson, Mona Persenius

**Affiliations:** ^1^ Department of Health Science Faculty of Health, Science and Technology Karlstad University Karlstad Sweden; ^2^ Faculty of Public Health Studies Inland Norway University of Applied Sciences Elverum Norway

**Keywords:** nursing staff, older people, oral care, perceptions, quality of care

## Abstract

**Aim:**

(a) To describe and compare perceptions of humanity aspects of oral care quality in relation to nursing staff in short‐term care units and intensive care units and older people in short‐term care units and their person‐related conditions; and (b) to compare humanity aspects of oral care quality perceptions between nursing staff and older people in short‐term care units.

**Design:**

Cross‐sectional study. Self‐reported questionnaire and clinical assessments.

**Methods:**

Nursing staff (*N = *417) and older people (*N = *74) completed the modified Quality of Care from a Patient Perspective instrument and person‐related items. Older people's oral health status was clinically assessed using the Revised Oral Assessment Guide. Data were analysed using descriptive and analytic statistics. The data were collected from 2013–2016.

**Results:**

Nursing staff's perceptions of humanity aspects of oral care quality were related to gender, work role and care environment. Older people's perceptions of humanity aspects of oral care quality were related to self‐reported physical health. Nursing staff in short‐term care units perceived the subjective importance of humanity aspects of oral care quality higher compared with older people in short‐term care units.

## 1 INTRODUCTION

Oral care quality has often focused on clinical dental indices, and while these provide valuable information, they might fail to recognize what is important for patients and nursing staff (National Institute for Health & care Excellence, 2016). Measures of oral care quality as a part of a patient's oral care experiences are twofold and can be classified as patient‐reported outcome measures (PROM) and as patient‐reported experience measures (PREM), the latter of which include assessments of perceptions and of satisfaction with care received (Kingsley & Patel, 2017). Meeting patients' needs, values and preferences might be seen as a critical aspect of healthcare performance (Docteur & Coulter, 2012; World Health Organization, 2015).

Today, most people will live into older age, and an increasingly significant proportion of the population will be older people (World Health Organization, 2015). As people age, they are more likely to experience several health conditions at the same time and thus increase the demands on different healthcare services such as intensive care and residential care (Nguyen, Angus, Boumendil, & Guidet, 2011; World Health Organization, 2015). Patients in intensive care units (ICUs) and in short‐term care (STC) units, a form of residential care facility (Dwyer, 2011), are not a homogeneous group, but they share many risk factors for impaired oral health (Drinka, 2010; Manabe, Teramoto, Tamiya, Okochi, & Hizawa, 2015) and the development of pneumonia (Sousa, Ferrito, & Paiva, 2018; Teramoto, Yoshida, & Hizawa, 2015). Optimal (Price, Duffy, & McCallum, 2015) oral care might prevent such adverse events in patients' oral health, physical health and psychological well‐being (Hua et al., 2016; Tada & Hanada, 2010). A recent study in ICUs and STC units that investigated Registered Nurses' (RNs), Enrolled Nurses' (ENs) and nurse assistants' (NAs) perceptions of oral care quality showed several areas for improvement (Andersson, Wilde Larsson, & Persenius, 2019). However, oral care quality cannot be understood fully without some appreciation, according to Hanefeld, Powell‐Jackson, and Balabanova (2017), of social norms, relationships, values and trust within the care environments where nursing care is provided.

## 2 BACKGROUND

Humanity highlights the importance of the relationship between patients and nursing staff (Donabedian, 1988; McCormack, 2003) and is underpinned by integrating the relational aspects and physical health and psychological well‐being (Edvardsson, 2015; Ricoeur, 1994). Humanity is one key attribute of quality (Beattie, Shepard, & Howieson, 2013; Hanefeld et al., 2017; World Health Organization, 2015), and it influences the extent to which patients experience quality care (Edvardsson, Watt, & Frances Pearce, 2017). The theoretical model of care quality from the patient perspective according to Wilde, Starrin, Larsson, and Larsson (1993) includes humanity aspects such as an identity‐oriented approach concerning information, respect and participation and a sociocultural atmosphere concerning the determination of needs and preferences. This means that humanity in oral care quality comprises nursing staff's ability to respect patients' needs and preferences, to include them in decision‐making and to provide information to enable self‐care (Coulter, 2011).

Humanity aspects of oral care quality might be influenced by person‐related conditions (e.g. age, gender, education, health conditions) (Donabedian, 1988; McCormack, 2003). Previous care quality studies in residential care and hospital environments showed that older women with higher self‐reported psychological well‐being (Grøndahl Abrahamsen & Fagerli, 2017; Grøndahl Abrahamsen, Karlsson, Hall‐Lord, Appelgren, & Wilde‐Larsson, 2011) and with lower level of education (Grøndahl Abrahamsen et al., 2011) were more likely to score the humanity aspects of care quality received higher than men (Grøndahl Abrahamsen & Fagerli, 2017; Grøndahl Abrahamsen et al., 2011) and younger men with higher education level (Grøndahl Abrahamsen et al., 2011). No relationship was found between physical health and perceptions of care quality received (Grøndahl Abrahamsen & Fagerli, 2017; Grøndahl Abrahamsen et al., 2011).

To our knowledge, no studies have investigated the relationship between nursing staff's person‐related conditions and their perceptions of oral care quality provided. However, relationships between nursing staff's person‐related conditions and their perceptions of care quality in general have been investigated in ICUs and residential care facilities (Hunter, Hadjistavropoulos, Thorpe, Lix, & Malloy, 2016; Rodriguez‐Martin, Stolt, Katajisto, & Suhonen, 2016; Stalpers, Linden, Kaljouw, & Schuurmans, 2017; Vassbø et al., 2019). Increased age of nursing staff seemed to be related to higher scores on care quality provided (Rodriguez‐Martin et al., 2016), but other studies showed no such relationship (Hunter et al., 2016; Vassbø et al., 2019). Female nursing staff scored care quality provided higher than men (Hunter et al., 2016), but other studies showed that gender had no influence on perceptions of care quality (Rodriguez‐Martin et al., 2016; Stalpers et al., 2017). One study from municipality care showed that lower education levels among nursing staff were related to higher scores on care quality provided (From, Nordström, Wilde‐Larsson, & Johansson, 2013), but other studies have shown no relationship between education level and care quality (Hunter et al., 2016; Rodriquez‐Martin et al., 2016; Stalpers et al., 2017; Vassbø et al., 2019).

Humanity aspects of oral care quality received or provided might also be influenced by the care environment (Donabedian, 1988; McCormack, 2003). Older patients' care quality perceptions have been shown to be positively related to nursing staff's satisfaction with care (Kvist, Voutilainen, Mäntynen, & Vehviläinen‐Julkunen, 2014), and patients tend to rate care quality received higher when nursing staff have a more person‐centred approach (Edvardsson et al., 2017). However, Grøndahl Abrahamsen et al. (2011) found that patients in hospitals scored the sociocultural atmosphere more negatively as the number of RNs in the units increased. The opposite was found in residential care, where a positive relationship was found between older people's perceptions of sociocultural atmosphere and the number of nursing staff in the units (Grøndahl Abrahamsen & Fagerli, 2017).

## 3 AIM

Little is known about nursing staff in ICUs and STC units and how their person‐related conditions and the care environment influence humanity aspects of perceptions of oral care quality. Knowledge is also lacking about older people in STC units with oral care dependency and how their person‐related conditions influence their perceptions of humanity aspects of oral care quality. Nursing staff's and older people's perspectives of care provided or received are important aspects when attempting to improve oral care quality, and it is important to get a deeper insight into what is important for older people and nursing staff so as to design and provide oral care with optimal quality. Therefore, the aim of this study was (a) to describe and compare humanity aspects of oral care quality perceptions in relation to nursing staff in short‐term care units and intensive care units and older people in short‐term care units and their person‐related conditions; and (b) to compare humanity aspects of oral care quality perceptions between nursing staff and older people in short‐term care units.

## 4 THE STUDY

### 4.1 Design

A cross‐sectional study was conducted involving a questionnaire with a sample of Swedish nursing staff in ICUs and STC units and of Swedish older people in STC units.

### 4.2 Setting

Participants were recruited from STC units and ICUs. An STC unit is a form of intermediate care concerned with a person's transition between hospital and home (Melis, Rikkert, Parker, & van Eijken, 2004). In Sweden, STC is a municipal effort to meet temporary healthcare needs of older people who need nursing care both day and night or support for older people waiting for care‐home placement, undergoing rehabilitation or requiring end‐of‐life care or when family members who are informal caregivers require recurrent relief (The National Board of Health & Welfare, 2013).

According to Marshall et al. (2017), the ICU is an organized system for the provision of care to critically ill patients which provides intensive and specialized medical and nursing care. The ICU has enhanced capacity for monitoring along with various methods for supporting vital organs and sustaining life during a period of life‐threatening organ system insufficiency (Marshall et al., 2017).

The STC units in the 19 municipalities were located in five counties in central and northern Sweden, and the ICUs were located in four of those counties. The municipalities represented both densely and sparsely populated regions, and the ICUs represented university hospitals (teaching hospitals that provide highly specialized care), county hospitals (those that provide general and to some extent specialized care) and local hospitals (those that provide general care).

RNs have an overall responsibility for ensuring quality nursing care in STC units and ICUs (Marshall et al., 2017; Swedish Society of Nursing, 2017). In Sweden, nursing studies are part of higher education and there are three years of education (bachelor's degree) to become a RN (Swedish Society of Nursing, 2017). RNs have licensure to diagnose, order and provide nursing care autonomously (Nursing & Midwifery Board of Australia, 2016a; Swedish Society of Nursing, 2017). ENs undertake a Diploma of Nursing (Nursing & Midwifery Board of Australia, 2016b), and in Sweden, ENs often have a postsecondary education in nursing and social services (The National Board of Health & Welfare, 2006). They work under the direct or indirect supervision of the RN as a part of the nursing care team and are responsible for their own actions in providing delegated nursing care (Nursing & Midwifery Board of Australia, 2016b; The National Board of Health & Welfare, 2013).

In STC units, RNs have a consulting role and ENs with a lower level of nursing education perform most bedside nursing care (Etherton‐Beer, Venturato, & Horner, 2013). This is in contrast to ICUs where RNs work with the patient at the bedside around the clock and continually oversee, coordinate and provide nursing care (Marshall et al., 2017) together with ENs (Falk & Wallin, 2016).

Nursing staff were recruited from 23 STC units in 19 municipalities and from six ICUs in four counties. The inclusion criteria were being employed as an RN or EN and working part‐time or full‐time. A total of 417 out of 814 nursing staff participated in the study, which represents a response rate of 51%. Table 1 presents the description of the nursing staff participants.

**Table 1:** Descriptions of nursing staff (*N = *417) in intensive care units (ICUs) and short‐term care (STC) units and their person‐related conditions

Person‐related conditions	Nursing staff		
	Total	ICUs	STC units
	*N* (%)	*N* (%)	*N* (%)
Participants	417	154 (37)	263 (63)
Age			
Mean (*SD*)	47.1 (11.11)	48.6 (11.57)	46.2 (10.74)
19–47	179 (45)	62 (41)	117 (48)
48–66	217 (55)	89 (59)	128 (52)
Missing	21	3	18
Gender			
Female	396 (96)	141 (92)	255 (97)
Male	18 (4)	12 (8)	6 (3)
Missing	3	1	2
Work role			
Registered Nurse	120 (40)	89 (58)	31 (12)
Enrolled Nurse	297 (60)	65 (42)	232 (88)

All older people admitted to the selected 23 STC units and another nine STC units in the 19 municipalities were eligible for study participation. The inclusion criteria were ≥65 years of age, having stayed 3 days or more in an STC unit, being dependent on help with oral care, understanding Swedish and having a health status—as estimated by the responsible RN in each STC unit—that allowed for participation and the ability to answer the questionnaire. A total of 74 out of 391 older people fulfilled the inclusion criteria, and they (*N = *74) all agreed to participate in the study. Table 2 presents a description of these participants.

**Table 2:** Descriptions of older people (*N = *74) in short‐term care (STC) units and their person‐related conditions

Person‐related conditions	Older people *N* (%)
Age	
Mean (*SD*)	83.6 (7.90)
68–79	24 (32)
80–85	20 (27)
85<	30 (41)
Gender	
Female	29 (39)
Male	45 (61)
Education	
Compulsory school	52 (71)
Upper secondary school	18 (25)
University	3 (4)
Self‐reported physical health	
Good to very good	32 (43)
Neither good nor poor	11 (15)
Poor to very poor	31 (42)
Self‐reported psychological well‐being	
Good to very good	39 (54)
Neither good nor poor	12 (16)
Poor to very poor	22 (30)
Missing	1
Self‐reported oral health	
Good to very good	44 (59)
Neither good nor poor	19 (26)
Poor to very poor	11 (15)
Oral health status	
Normal	—
Moderate problems	52 (70)
Severe problems	22 (30)

The study part concerning older people and nursing staff in STC units was based on data from a larger research project (SOFIA) whose aim was to explore different aspects related to oral health in older people staying in STC units (Andersson et al., 2019; Andersson, Wilde‐Larsson, Carlsson, & Persenius, 2018; Hägglund et al., 2017). In the larger project, the questionnaire package contained six instruments, and two of those were used in this study.

### 4.3 Procedures

Data for nursing staff were collected in STC units from October 2013–January 2016 and in ICUs from January 2016–February 2016. Research assistants (seven registered dental hygienists and two doctoral students) in STC units and nurse managers or RNs appointed by nurse managers in ICUs gave information verbally to the nursing staff. Each nursing staff member was given a questionnaire with an addressed and prepaid envelope. Along with the questionnaire, nursing staff received written information about the study. Completed questionnaires in STC units were returned to the responsible researcher in the SOFIA project, and completed questionnaires in ICUs were returned to the first author (MA).

Data for older people in STC units were collected from October 2013–January 2016. The research assistants gave older people in STC units who fulfilled the inclusion criteria both verbal and written information about the study. Older people answered the modified version of the *Quality of Care from a Patient Perspective* (QPP) questionnaire (Wilde Larsson & Larsson, 2002) and the self‐reported physical health, self‐reported oral health condition and self‐reported psychological well‐being questions. The research assistants carried out clinical assessments of the older people's oral health status, and completed questionnaires were returned to the responsible researchers (SOFIA).

### 4.4 Measurements

#### 4.4.1 Humanity aspects of oral care quality

Nursing staff's and older people's perceptions of the humanity aspects of oral care quality were measured with the modified QPP questionnaire. The QPP questionnaire (Wilde Larsson & Larsson, 2002) is considered a high‐standard instrument with excellent validity and reliability (Beattie, Murphy, Atherton, & Lauder, 2015).

The modified QPP version directed towards nursing staff (Andersson et al., 2019) and older people (Andersson et al., 2018) included two QPP dimensions related to humanity. The QPP dimension of identity‐oriented approach (ID) included information (one item), oral care/oral health experience (one item), respect (one item), commitment (one item) and participation (one item). The QPP dimension of social–cultural atmosphere (SC) included the determination of needs and requests (one item). The total number of items was six (Table 3).

**Table 3:** Description of participants' perceptions of humanity aspects of oral care quality including perceived oral care reality (PR) and subjective oral care importance (SI)

Humanity aspects of oral care quality	Nursing staff (*N = *417)	Older people (*N = *74)
	PR/SI	PR/SI
	*N* (%)	*N* (%)
Dimension: Identity‐oriented approach with items		
The best possible information about oral care		
Optimal oral care quality	122 (32)/308 (80)	24 (39)/34 (54)
Suboptimal oral care quality	257 (68)/75 (20)	38 (61)/29 (46)
Understand how I/older people experienced their oral health		
Optimal oral care quality	214 (54)/355 (90)	33 (58)/41 (66)
Suboptimal oral care quality	182 (46)/41 (10)	24 (42)/21 (34)
Respectful towards me/older people in connection with oral care		
Optimal oral care quality	371 (91)/390 (96)	60 (88)/62 (90)
Suboptimal oral care quality	38 (9)/18 (4)	8 (12)/7 (10)
Showed commitment, cared about me/older people and my oral care/their oral care		
Optimal oral care quality	266 (65)/374 (92)	40 (64)/45 (65)
Suboptimal oral care quality	144 (35)/34 (8)	23 (36)/24 (35)
Good opportunity to participate in the decisions that applied to my/their oral care		
Optimal oral care quality	178 (47)/296 (79)	26 (52)/31 (58)
Suboptimal oral care quality	200 (53)/80 (21)	24 (48)/22 (42)
Dimension: Sociocultural atmosphere with item		
Oral care determined by my/their own requests and needs rather than nursing staff's procedures		
Optimal oral care quality	219 (54)/341 (86)	32 (46)/34 (68)
Suboptimal oral care quality	186 (46)/55 (14)	37 (54)/22 (32)

Note

Optimal oral care quality; PR = agree to completely agree. SI = high to very high subjective importance.

Suboptimal oral care quality; PR = not agree to partly agree. SI = quite high or no subjective importance.

Each item was evaluated in terms of perceived oral care reality (PR) and subjective oral care importance (SI). Each item's PR described how nursing staff and older people experienced various oral care aspects and thus reported their perceptions of actual care. Each item's SI described how important nursing staff and older people considered various aspects of oral care to be and thus reported their preferences.

The PR items were responded to on a four‐point Likert‐type scale as one (do not agree at all), two (partly agree), three (agree) and four (completely agree). The SI items were responded as one (little or no importance), two (quite high importance), three (high importance) and four (very high importance). Each PR and SI item also had a “non‐applicable” response (Wilde Larsson & Larsson, 2002). The mean value in each dimension was calculated by adding the item scores and dividing by the number of items answered. Items with “non‐applicable” responses were excluded in the calculation.

In the present study, Cronbach's alpha coefficients in the QPP dimension ID for nursing staff in STC units and ICUs were 0.78 for PR and 0.82 for SI, and for older people in STC units, they were 0.88 for PR and 0.83 for SI.

### 4.5 Person‐related conditions and care environment

Nursing staff's person‐related conditions were assessed by age, gender and work role. Care environment was measured using type of unit (ICU or STC unit). Older people's person‐related conditions were assessed by age, gender, education, self‐reported physical health, oral health condition, psychological well‐being and assessment of oral health status using the *Revised Oral Assessment Guide* (ROAG) (Andersson, Hallberg, & Renvert, 2002). ROAG includes assessments of nine items—voice, lips, mucous membranes, tongue, gums, teeth, dentures, saliva and swallowing—scored as normal, moderate oral health problem or severe oral health problem (Andersson et al., 2002).

### 4.6 Analysis

Humanity aspects of oral care quality including the QPP dimensions ID and SC, person‐related conditions and care environments were examined with descriptive statistics.

When comparing the mean scores of the QPP dimensions ID and SC, between care environment and person‐related condition, Student's *t* test was used when comparing two groups. One‐way analysis of variance (ANOVA) was used when comparing three groups. Statistically significant interactions in the ANOVA were followed by Tukey's test for post hoc comparisons to analyse differences in mean values between groups (Pallant, 2013; Polit & Beck, 2012). Statistical significance was set at *p* < .05. When small groups (*N* ≤ 20) were involved, the alpha level was adjusted to *p* < .10 (Pallant, 2013). Cronbach's alpha was used to test internal consistency (Pallant, 2013). SPSS version 24.0 (IBM Inc.) was used for all analyses.

### 4.7 Ethics

Nursing staff and older people were informed verbally and in writing that their participation was voluntary and that their identity would be kept confidential. Older people were free to withdraw from the study at any time without explanation. The research assistants obtained older people's consent to take part in the study in writing and returned it to the responsible researchers (SOFIA). The Regional Ethical Review Board in Uppsala, Sweden, approved the study for STC units (Dnr 2013/100) and for ICUs (Dnr 2015/457).

## 5 RESULTS

### 5.1 Person‐related conditions of the participants

#### 5.1.1 Nursing staff

The mean age of the nursing staff was 47 years. Most participants (96%) were women, 60% were ENs, and 63% of the nursing staff worked in STC units (Table 1).

#### 5.1.2 Older people

The mean age of the older people was 84 years, and 61% were male. In terms of education status, 71% had compulsory school as their highest level of education. The older people's self‐reported physical health conditions varied, with 43% reporting that they had good or very good physical health and 42% reporting that they had poor or very poor physical health. Fifty‐nine per cent of older people reported that their oral health was good to very good, despite the fact that 70% of them were clinically assessed to have moderate oral health problems and 30% to have severe oral health problems. Good‐to‐very good psychological well‐being was reported by 54% and poor‐to‐very poor psychological well‐being by 30% (Table 2).

### 5.2 Participants' perceptions of humanity aspects of oral care quality

Perceptions of humanity aspects of oral care quality, including QPP dimensions ID and SC, were interpreted as follows. PR scores of agree to completely agree (≥3) were interpreted as optimal oral care quality, and PR scores of not agree to partly agree (<3) were interpreted as suboptimal oral care quality. SI scores of high or very high importance (≥3) were interpreted as high subjective importance for oral care quality, and SI scores of little or no importance (<3) were interpreted as low importance for oral care quality.

#### 5.2.1 Nursing staff

Most (91%) of nursing staff scored optimal oral care quality (PR) on the QPP dimension ID and on the PR item related to respect towards older people/patients in connection with oral care. Respect was also the item that 96% perceived as having high SI for oral care quality. The highest proportion (68%) of nursing staff scored suboptimal oral care quality (PR) on the QPP dimension ID and on the PR item concerning best possible oral care information. Providing the patient/older person with the opportunity to participate in oral care was the item most commonly scored (21%) by the nursing staff to have low SI for oral care quality.

Fifty‐four per cent of nursing staff perceived optimal oral care quality (PR) on the QPP dimension SC and on the PR item concerning determination of oral care based on older people's needs and requests. Eighty‐six per cent perceived that this was of high SI for oral care quality (Table 3).

The range “non‐applicable” responses in PR and SI scales for nursing staff, were mostly between on to four, except items related to information and participation. The item information had 23 “non‐applicable” responses in PR and 17 “non‐applicable responses” in SI. The item participation had 29 “non‐applicable” responses in PR and 23 “non‐applicable” responses in SI.

#### 5.2.2 Older people

On the QPP dimension ID and the PR item concerning respect, 88% of older people reported optimal oral care quality (PR) and 90% perceived respect to have high SI for oral care quality. Sixty‐one per cent of older people perceived suboptimal oral care quality (PR) concerning PR item best possible oral care information, and 46% perceived such information to have low SI for oral care quality.

On the QPP dimension SC and the PR item concerning determination of oral care based on their needs and requests, 46% of older persons reported optimal oral care quality (PR) and 68% perceived that this was of high SI for oral care quality (Table 3). No “non‐applicable” responses for older people were found in PR scale or in SI scale.

### 5.3 Comparisons between participants' perceptions of humanity aspects of oral care quality and person‐related conditions and care environment

The SI on the QPP dimension ID was statistically significantly higher among women, ENs and nursing staff working in STC units compared with men, RNs and those working in ICUs. There were no statistically significant differences for nursing staff's perceptions of PR on the QPP dimension ID in relation to age, gender, work role or care environment.

The QPP dimension SC's PR and SI mean values were statistically significantly higher among ENs compared with RNs. The SI mean value in the dimension was also statistically significantly higher among nursing staff working in STC units compared with nursing staff working in ICUs. There were no statistically significant differences in the QPP dimension SC's PR mean value in relation to age, gender or care environment or in the SI mean value in relation to age or gender (Table 4).

**Table 4:** Comparison of nursing staff's perceptions of humanity aspects of oral care quality, including perceived oral care reality (PR) and subjective oral care importance (SI), in relation to person‐related conditions and care environment

Person‐related conditions	Humanity aspects of oral care quality							
	Identity‐oriented approach dimension				Sociocultural atmosphere dimension			
	PR		SI		PR		SI	
	Mean (*SD*)	*p*‐Value	Mean (*SD*)	*p*‐Value	Mean (*SD*)	*p*‐Value	Mean (*SD*)	*p*‐Value
Age								
19–47	2.64 (0.528)	.318	3.33 (0.491)	.852	2.56 (0.930)	.779	3.27 (0.775)	.685
48–66	2.70 (0.625)		3.32 (0.542)		2.59 (0.895)		3.30 (0.678)	
Gender								
Female	2.69 (0.586)	.368	3.34 (0.504)	**.030^a^**	2.58 (0.920)	.947	3.28 (0.740)	.871
Male	2.54 (0.634)		3.04 (0.608)		2.59 (0.618)		3.25 (0.577)	
Work role								
Registered Nurse	2.61 (0.599)	.146	3.17 (0.553)	**<.0001**	2.36 (0.903)	**.002**	3.16 (0.753)	**.036**
Enrolled Nurse	2.71 (0.581)		3.39 (0.497)		2.67 (0.897)		3.33 (0.722)	
Care environment								
Short‐term care unit	2.64 (0.585)	.111	3.38 (0.504)	**.007**	2.61 (0.854)	.3	3.33 (0.685)	**.041**
Intensive care unit	2.76 (0.586)		3.22 (0.544)		2.51 (0.993)		3.17 (0.808)	

Note

PR scale ranges from 1 (do not agree at all)–4 (completely agree). SI scale ranges from 1 (little or no importance)–4 (very high importance). Statistical analysis = Student's *t* test. Statistical significance at *p* < .05 and *p* < .10^a^.

Bold indicates statistical significant value.

On the QPP dimension SC, older people with a self‐reported physical health condition (small group) of good to very good scored a SI mean value of 3.10 (0.77), which was statistically significantly higher (*p* = .065) compared with older people with a self‐reported physical health condition of neither good nor poor who scored a SI mean value of 2.40 (1.17) (not shown in table).

Comparison of older people's perceptions of humanity aspects of oral care quality on the QPP dimensions ID (PR and SI) and SC (PR and SI) in relation to age, gender and education showed no statistically significant differences. There were also no statistically significant differences between the QPP dimension's PR and SI in relation to self‐reported physical health, oral health condition or psychological well‐being (not shown in table).

### 5.4 STC units: comparisons between nursing staff's and older people's perceptions of humanity of oral care quality

The mean values for SI in humanity aspects of oral care quality, including QPP dimension ID and SC, were statistically significantly higher among nursing staff compared with older people. For PR, no statistically significant differences were found in humanity aspects of oral care quality between nursing staff and older people in STC units (Table 5).

**Table 5:** Comparison of humanity aspects of oral care quality perceptions, including perceived oral care reality (PR) and subjective oral care importance (SI), between nursing staff and older people in short‐term care units

Humanity aspects of oral care quality	Nursing staff	Older people	*p*‐Value
	*N = *263	*N = *74	
Dimensions	Mean (*SD*)	Mean (*SD*)	
Identity‐oriented approach			
PR	2.65 (0.585)	2.83 (0.846)	.216
SI	3.38 (0.504)	2.88 (0.681)	**<.0001**
Sociocultural atmosphere			
PR	2.61 (0.854)	2.57 (1.182)	.745
SI	3.33 (0.685)	2.87 (0.862)	**<.0001**

Note

PR scale ranges from 1 (do not agree at all)–4 (completely agree). SI scale ranges from 1 (little or no importance)–4 (very high importance). Statistical analysis = Student's *t* test. Statistical significance at *p* < .05. 1

## 6 DISCUSSION

### 6.1 Nursing staff

Perceptions of humanity aspects of oral care quality including QPP dimensions ID and SC (e.g. information, participation and determination of needs and preferences) showed that women and ENs and those working in STC units scored the humanity aspects of oral care quality, including both PR and SI, higher compared with men, RNs and those working in ICUs.

Intensive care is primarily aimed to saving lives, and the application of life‐sustaining technology, advanced treatment and close observations (Marshall et al., 2017) might lead to objectifying and depersonalizing the patient (McLean, Coombs, & Gobbi, 2016). Many patients in ICUs are comatose, sedated or in other ways affected by life‐threatening illnesses, and active patient participation in nursing care and treatment might be difficult to achieve (Schandl, Falk, & Frank, 2017). However, patients who are awake during critical illness and mechanical ventilation have reported experiencing a sense of vulnerability (Engström, Nyström, Sundelin, & Rattray, 2013; Karlsson, Bergbom, & Forsberg, 2012; Laerkner, Egerod, Olesen, & Ploug Hansen, 2017) and a desire to participate in communication and in their own care as soon as they perceived they could (Karlsson et al., 2012; Laerkner et al., 2017; Lindberg, Sivberg, Willman, & Fagerström, 2015).

Participation in their nursing care has been shown to make the patients feel better and to start to believe in their recovery (Karlsson et al., 2012).

Identity‐oriented aspects such as participation and information might decrease fear and insecurity and improve the recovery process for patients in ICUs (Wassenaar, Schouten, & Schoonhoven, 2014). A recent study in ICUs (Andersson et al., 2018) showed that most ICU nurses perceived that they involved patients in oral care when patients were able to participate. Nursing staff might perceive patient involvement as challenging because of the patients' health conditions (Falk, Schandl, & frank, C., 2019; Olding et al., 2016) and because of the prioritizing of medical tasks and measures (Falk et al., 2019). During life and death situations, nursing staff's lower level of identity‐oriented approaches might in fact be a necessity for delivering safe and effective nursing care (McLean et al., 2016). However, ICU patients' needs and preferences of participation remain central for the humanity aspects of oral care quality. To what extent the care environment is focused on person‐centredness (Edvardsson, Sandman, & Rasmussen, 2009) in ICUs is to our knowledge lacking, and thus, there is a need for further investigation in this regard.

In the present study, ENs scored humanity aspects of oral care quality as more important compared with RNs. This might be because ENs in STC units, in contrast to the RNs in STC units, are more frequently involved in direct care for older people (Etherton‐Beer et al., 2013; Hewko et al., 2015). This is in line with a previous study where it was discussed that RNs might be too distant in residential care to have control over, assess and critically judge the quality of care (From et al., 2013). The care environment in ICUs is the opposite, with a high density of RNs who continually oversee, coordinate and provide nursing care (Falk & Wallin, 2016; Marshall et al., 2017). It is unclear why RNs in ICUs scored humanity aspects of oral care quality lower compared with ENs in ICUs, and this needs further investigation.

### 6.2 Older people

Older people scored the humanity aspects of oral care quality to be suboptimal in terms of both PR and SI. Older people with higher self‐reported physical health considered it more important that oral care was based on their own needs and preferences compared with those with lower self‐reported physical health.

Previous studies have shown that older patients' preferences for involvement in decision‐making differ and that some of them prefer a more active role than others (Kiselev, Suija, Oona, Mellenthin, & Steinhagen‐Thiessen, 2018; Wiltjer, 2019), but physical health might have an impact on the degree to which older patients want to or are able to participate in their own care (Kiselev et al., 2018). Lack of energy and ability among some older patients to participate in their own care has been previously been reported due to illness, and difficulties in accomplishing even minor tasks are one reason for not participating in nursing care (Nyborg, Kvigne, Danbolt, & Kirkevold, 2016; Ringdahl, Chaboyer, Ulin, Bucknall, & Oxelmark, 2017). An adapted and re‐evaluated view of oral health and oral care might occur (Brondani, 2010; Custers, Westerhof, Kuin, Gerritsen, & Riksen‐Walraven, 2013) based on patients' decisions to use their energy in other ways than maintaining their oral health (Andersson et al., 2018; Niesten, Mourik, & Sanden, 2013).

### 6.3 In STC units: comparison between nursing staff and older people

The comparison between nursing staff and older people's perceptions showed differences in SI of humanity aspects where nursing staff scored the SI of humanity aspects of oral care quality higher compared with older people.

The humanity aspect of oral care quality was perceived by nursing staff and older people to be suboptimal. Information is important to be able to participate, and information might help older people to perceive themselves as being involved in their own care (Xie, Wang, Feldman, & Zhou, 2012). It is also important that older people share information (Nyborg et al., 2016; Ringdahl et al., 2017), but they might not provide any information to nursing staff unless the nursing staff asks for it (Nyborg et al., 2016). In the present study, the nursing staff perceived that respect towards older people was important, and this is in line with previous studies (Coker, Ploeg, Kaasalainen, & Carter, 2017; Ek, Browall, Eriksson, & Eriksson, 2018). Nursing staff have reported not pursuing oral care further with older people with care dependency who decline help with oral care, and nursing staff have also reported a reluctance to ask an older person's permission to inspect their oral cavity (Coker et al., 2017; Ek et al., 2018). Nursing staff's perceived respect in combination with older people's passive stance towards information and their perceptions of the lower importance of humanity aspects of oral care quality might lead to undiscovered oral health problems among older people with declining physical health.

### 6.4 Strengths and limitations

It should be noted that the selected person‐related conditions in the present study did not cover all aspects; for example, culture, socioeconomics or nursing staff's education in oral health and the care environment only covered the type of unit. Care environment aspects of interest could have been, for example, number of RNs in the units, how nursing care was organized, how many beds in the units and average length of patients/older people stay in the units.

All nursing staff and older people who met the inclusion criteria were invited to participate, and the response rate for nursing staff was 51%. Due to incomplete data about nursing staff's person‐related conditions in STC units, no analysis of dropouts could be performed. There might be several reasons why nursing staff in this study chose not to participate. For example, high workloads might have had a negative impact on participants' ability to answer the questionnaire, or participants might have considered the study subject to not be relevant to them (Morton, Bandara, Robinson, & Atatoa Carr, 2012).

There were a greater number of STC units where older people participated in the study than the number of STC units where nursing staff participated. The explanation for this is that the nursing staff worked in more than one STC unit, but they indicated the unit where they spent most of their working time.

The internal consistency of the modified version of QPP was acceptable. The modification of the questionnaire is based on a selection of items from the short version of QPP (Wilde Larsson & Larsson, 2002). The short version of the QPP dimension SC contained four items (Wilde Larsson & Larsson, 2002), but only one item was considered relevant for the purpose of this study. However, one SC item reflects the theoretical model of care quality (Wilde et al., 1993), but more aspects might be necessary for the understanding of oral care and SC. To secure construct validity, the modified version of QPP needs to be further psychometrically tested.

More nursing staff in ICUs than nursing staff in STC units gave “non‐applicable” responses about information and participation. An explanation might be that the nursing staff in ICUs might not see active patient participation as possible because of patients' life‐threatening health conditions.

Responsible RNs in each STC unit decided which older people to ask to participate based on their health status and their ability to answer the questionnaire. The RNs might have had somewhat different views of an older person's health condition, and according to Polit and Beck (2012), this is a possible threat to the internal validity.

The self‐reported questionnaire might have been challenging for older people with limited functional capacity. If necessary, the research assistants helped older people by filling in the questionnaire based on the person's answers to each item. This could have influenced older people's oral care quality perceptions (Wilde Larsson, 2000). However, the research assistants were not involved in the participants' care, so the older people were not in any form of dependence with regard to the research assistants (World Medical Association, 2019).

Statistically significant differences are mainly dependent on the number of participants and larger samples have smaller samplings errors, but in quantification of differences, it is important to distinguish clinical significance from statistical significance (Angst, Aeschlimann, & Angst, 2017; Polit, 2017). The sample sizes in certain group levels (gender for nursing staff and age, self‐reported physical health, oral health and psychological well‐being for older people) were small, and thus, the significance level was adjusted to reduce the risk for type II errors due to insufficient power (Pallant, 2013). Despite the measures taken, the results should be interpreted with caution, and further studies with larger samples need to be performed.

Statistical significance, however, does not guarantee that results will have clinical significance, and for drawing conclusions about clinical significance at group levels, values might be stipulated (Polit, 2017). In the present study, the participants' responses “agree” to “completely agree” (PR scale) were stipulated as optimal oral care quality. Participants' responses “not agree” to “partly agree” (PR scale) were stipulated as suboptimal.

In the present study, there were several statistical significances between participants' perceptions of humanity aspects of oral care quality and person‐related conditions. Despite small differences in several mean scores, the results might be considered as clinically relevant. The knowledge of the variation in participants' perceptions according to person‐related conditions might help RNs and ENs to be aware of older people needing special attention, thereby enhancing provision of person‐centred care. The awareness of differences might also increase the commitment for improving oral care because according to Stelson, Hille, Eseonu, and Doolen (2017), the nursing staff perceive changes as being necessary.

The ICU represented different sizes in four different regions, and the STC units represented municipalities from both densely and sparsely populated regions in Sweden. Perception variations about humanity aspects of oral care quality might be considered representative to nursing staff and make the present results generalizable to similar staffing arrangements. Participating older people in the present study represent a variety of reasons for being admitted to STC units. Common for the older people is that they all share the temporary need for oral care. The distribution of the older people among the different STC units was between 1–8 participants per STC units and might reflect older people who receive STC in Sweden and their perceptions about humanity aspects of oral care quality.

## 7 CONCLUSION

Despite differences between and within organizations of oral care across ICUs and STC units, all healthcare services should deliver oral care according to older people's needs and preferences. Most nursing staff perceived that insufficient attention was given to humanity aspects of oral care that they felt was important. However, they might not be able to convert intentions into practice unless the older person asks for it. It seems that nursing staff provide optimal oral care that to a higher degree acknowledges the needs and preferences of older people with higher self‐reported physical health. They might not always provide optimal quality oral care for older people with declining health conditions.

## CONFLICTS OF INTEREST

The authors declare no conflict of interest.

## Footnotes

1 Bold indicates statistical significant value.
